# Ayahuasca potential benefits

**DOI:** 10.1192/j.eurpsy.2021.2043

**Published:** 2021-08-13

**Authors:** R. Pinilla, C. Rodriguez, D. Batet-Sanchez, B. Ordoñez

**Affiliations:** Psiquiatria, Hospital de Getafe, Madrid, Spain

**Keywords:** ayahuasca, therapeutic, neuropharmacology

## Abstract

**Introduction:**

Ayahusca has potential therapeutic beneffits.

**Objectives:**

Expose the potential beneffits of ayahuasca from neuropharmacology and clinical existing evidence.

**Methods:**

A literature review was carried out in the databases pubmed, clinical key and texts of scientific dissemination.

**Results:**

There´s scientific literature about the potential therapeutic use of ayahuasca in dependencies, anxiety symtoms and depression, near death experiences and terminal illnesses. Possible benefit is postulated in impulsivity and personality disorders. It induces an introspective state, triggered by thoughts, emotions and autobiographical memories, which promotes reflection on personal issues, allowing new perspectives on certain life issues. It is common for users to describe it as analogous to a psychotherapeutic intervention. 5HTA2 agonists stimulate the expression of genes that encode transcription factors such as c-fos, egr 1, egr 2 and brain-derived neurotrophic factor (BDNF), which influence neuronal plasticity and are associated with cognitive aspects such as memory and attention. MAOIs and 5HT2A agonism have anxiolytic and antidepressant effects. Sigma -1 agonism promotes neuroplasticity. Decrease and remission in the consumption of alcohol and cocaine has been reported in patients with abuse and dependence. There has been significant decrease in depressive symptomatology, in observational studies, cases and controls and double blind compared with placebo. Improvement in different domains measured with mindfulness scales, similar to those observed in meditators, suggests an association between mindfulness techniques and experiences with ayahuasca.

**Conclusions:**

There is existing evidence about potential therapeutical uses of ayahuasca. More studies are needed with biger samples, to establish it´s clinical use.

**Disclosure:**

No significant relationships.

